# Transcatheter arterial chemoembolization combined with CT-guided percutaneous thermal ablation versus hepatectomy in the treatment of hepatocellular carcinoma

**DOI:** 10.1186/s40880-015-0023-9

**Published:** 2015-06-10

**Authors:** Sheng Li, Liang Zhang, Zhi-Mei Huang, Pei-Hong Wu

**Affiliations:** Department of Medical Imaging & Interventional Radiology, Sun Yat-sen University Cancer Center; State Key Laboratory of Oncology in South China; Collaborative Innovation Center for Cancer Medicine, Guangzhou, Guangdong 510060 People’s Republic of China

**Keywords:** Percutaneous thermal ablation, Transcatheter arterial chemoembolization, hepatectomy, Hepatocellular carcinoma, Survival

## Abstract

**Introduction:**

Transcatheter arterial chemoembolization (TACE) plus thermal ablation has been widely used recently in the treatment of hepatocellular carcinoma (HCC). In this study, we aimed to compare results of the combination of TACE and percutaneous thermal ablation with those of hepatectomy in patients with HCC.

**Methods:**

The clinical data of 137 HCC patients who sequentially received TACE and computed tomography (CT)-guided percutaneous thermal ablation as an initial curative treatment (combination group) and 148 matched HCC patients who received hepatectomy (surgery group) between 2004 and 2011 were collected and analyzed. After TACE, multiphase contrast-enhanced CT was performed to identify the total number of tumors as well as lipiodol deposition in the liver. Survival was calculated by using the Kaplan-Meier method and compared by using the log-rank test. The prognostic factors were assessed with multivariate Cox proportional hazards regression analysis.

**Results:**

Of all 285 patients, 225 (79.0 %) had cancerous lesions ≤ 5 cm in diameter. In preoperative contrast-enhanced CT or magnetic resonance imaging, the number of tumors was 1–4 for each patient. The 1-, 3-, and 5-year overall survival rates were 95, 74 %, and 67 % in the combination group and 88, 66, and 47 % in the surgery group, respectively (*P* = 0.004); the corresponding recurrence-free survival rates for the two groups were 92, 69, and 61 % and 75, 58, and 44 %, respectively (*P* = 0.001). In the multivariate analysis, treatment allocation was an independent prognostic factor for survival. Only 60 patients in the combination group had sufficient imaging data, and 135 new lesions with lipiodol deposition were diagnosed as malignancies in 22 of 60 patients, whereas 20 new lesions were found in 11 of 148 patients in the surgery group.

**Conclusion:**

The combination of TACE and CT-guided percutaneous thermal ablation for HCC improves survival of HCC patients compared with hepatectomy.

## Background

Hepatocellular carcinoma (HCC) is one of the most common cancers and is a primary contributor to cancer-related death in the world; new treatment modalities are needed to improve the survival of HCC patients [1–3].

Although hepatectomy is an effective treatment for HCC, it is also invasive and not always repeatable. For HCC smaller than 2 cm in diameter, radiofrequency ablation (RFA) achieved the same efficacy as surgery [4]. As a preoperative examination, lipiodol computed tomography (CT) also contributes to the detection of additional liver lesions after transcatheter arterial chemoembolization (TACE) [5–7].

The combination of TACE and RFA improves the survival of HCC patients compared with RFA alone [8]. However, few studies have compared the results of TACE plus thermal ablation with those of hepatectomy in patients with comparable characteristics [9]. In this retrospective study, we aimed to investigate the long-term outcomes of TACE combined with CT-guided percutaneous thermal ablation and hepatectomy as the initial curative treatment for resectable HCC.

## Patients and Methods

### Patient selection and patient characteristics

This comparative study collected and retrospectively analyzed the patient characteristics from our electronic medical database. The study was approved by the Institutional Review Board of Sun Yat-sen University Cancer Center.

Contrast-enhanced CT and magnetic resonance imaging (MRI) are the most common imaging techniques for the diagnosis of HCC. The number of tumors and tumor size were comparable between the two groups, as observed via preoperative imaging. The tumor number and tumor size were assessed by using contrast-enhanced CT or MRI prior to TACE or hepatectomy; however, additional HCCs that were detected via lipiodol CT or pathologic examination were not included. Dynamic contrast-enhanced CT was performed with a 16-detector row scanner (Brilliance 16, Philips Medical Systems, Best, the Netherlands) or a 64-detector row CT scanner (Brilliance 64, Philips Medical Systems, Best, the Netherlands). MRI examination was performed with a 1.5-T MR system (Magnetom Symphony, Siemens, Erlangen, Germany) with an 18-channel body surface phased-array coil. MRI scan was also performed with a 3 T whole-body scanner (Tim Trio, Siemens, Erlangen, Germany) in some patients by using the body coil to transmit radiofrequency pulses and a 12-channel phased-array head coil to receive signals. The liver was imaged in at least the axial plane in all patients, and the gadoxetic acid (Primovist, Bayer Schering Pharma, Germany) was administrated at a dose of 0.1 mL/kg through the antecubital vein.

The diagnosis of HCC was based on the criteria used by the European Association for the Study of the Liver [10]: (1) two imaging techniques showing typical features of HCC, (2) positive findings on one imaging study together with an alpha fetoprotein (AFP) level of > 400 ng/mL, or (3) a histological diagnosis of HCC.

The inclusion criteria were as follows: (1) patients at 18–75 years old; (2) patients with HCC of 1–10 cm in diameter and 1–4 lesions in preoperative imaging (prior to TACE or surgery); (3) patients with Eastern Cooperative Oncology Group performance score of 0 or 1; (4) patients with liver function Child-Pugh A or B; and (5) patients without portal vein tumor thrombus, lymph node metastases, or distant metastases.

The exclusion criteria were as follows: (1) patients with other malignancies; (2) patients with liver function Child-Pugh C; (3) patients with less than 3 months of life expectancy or follow-up; (4) patients with uncontrolled severe diabetes, acute infection, or allergy to iodine; or (5) patients with a bleeding tendency (prothrombin time-international normalized ratio [PT-INR] > 2.5 or platelet < 50 × 10^9^) or severe jaundice (total bilirubin > 170 μmol/L).

**Fig. 1:**
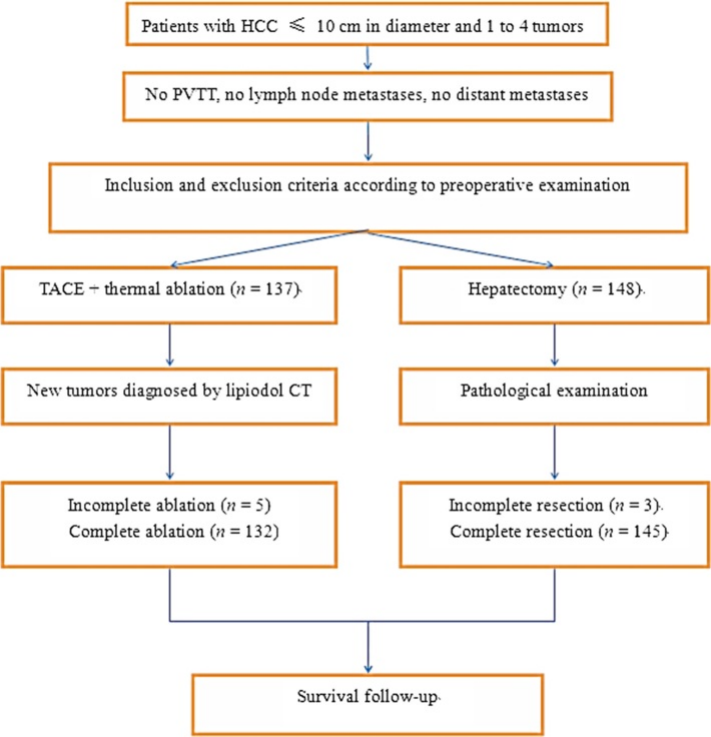
Chart showing the treatment strategy of hepatocellular carcinoma (HCC). PVTT, portal vein tumor thrombus; TACE, transcatheter arterial chemoembolization; CT, computed tomography

A total of 137 patients who received TACE and percutaneous thermal ablation (combination group) as well as 148 patients who received hepatectomy (surgery group) between January 2004 and December 2011 were involved (Fig. 1). The patients in the surgery group were selected from more than 10,000 HCC patients undergoing hepatectomy to match the patients in the combination group and reduce confounding factors. The patients in the two groups were matched according to age, sex, tumor size, tumor number, liver function, and other factors prior to TACE or surgery (Table 1).

**Table 1:** Comparison of the clinical parameters between the two groups of patients with hepatocellular carcinoma (HCC)

Matched variable	Combination group (*n* = 137)	Surgery group (*n* = 148)	*P*
Age (years)			0.093
Median	51	54	
Range	23–74	23–75	
Sex^a^			0.183
Male	129	133	
Female	8	15	
Hypersplenism^a^			0.281
Yes	74	70	
No	63	78	
Child-Pugh class^a^			0.193
A	124	140	
B	13	8	
Cirrhosis^a^			0.432
Yes	118	132	
No	19	16	
Diabetes mellitus^a^			0.296
No	118	134	
Yes	19	14	
Tumor number^a^			0.582
1	59	69	
2	43	50	
3	28	21	
4	7	8	
Tumor size^a^			0.992
≤3 cm	61	68	
>3 cm and ≤5 cm	47	49	
>5 cm and ≤7 cm	23	24	
>7 cm and ≤10 cm	6	7	
Alpha fetoprotein^a^			0.292
≥400 ng/mL	34	45	
<400 ng/mL	103	103	
Size of all nodules in diameter (cm)			0.364
Median	3.8	4.0	
Mean ± standard deviation	37.0 ± 17.5	38.9 ± 18.1	
Albumin (g/L)	40.8 ± 4.3	41.6 ± 4.2	0.133
Prothrombin time (s)	12.6 ± 2.0	13.0 ± 2.1	0.075
Platelet count (×10^9^/L)	128.2 ± 64.8	145.1 ± 60.0	***0.022***
Hemoglobin (g/L)	135.9 ± 16.2	138.7 ± 16.8	0.156
CA19-9 (U/mL)	140.4 ± 852.2	79.8 ± 531.0	0.469

^a^The values are presented as number of patients. Tumor size, the largest lesion in diameter.

### Treatments

#### TACE and lipiodol CT

TACE was performed prior to ablation and by following previously described techniques [11]. The volume of lipiodol and the dose of chemotherapeutics depended on the volume and blood supply of the targeted tumor, as well as the Eastern Cooperative Oncology Group (ECOG) performance status and liver function of the patients. The emulsion of 5–15 mL of lipiodol, 40–60 mg of epirubicin, 4 mg of mitomycin C, 200–400 mg of carboplatin, and 3 mL of saline solution was infused through the target hepatic arteries, without retrograde flow. Two weeks to 1 month after TACE, baseline CT and multiphase (arterial, portal, and delayed phases) contrast-enhanced CT (lipiodol CT) were performed to identify the total number of tumors as well as lipiodol deposition in the liver. We then treated all confirmed cancerous lesions. The tiny HCC was defined as tumors ≤ 5 mm in diameter.

For lipiodol deposition in the liver, we first excluded calcification, intrahepatic calculi, hemangioma, patchy lipiodol, hypervascular focal nodular hyperplasia, and satellite lesions within the primary lesion. Tiny HCC with lipiodol deposition was confirmed by at least two radiologists in our department who each had more than 4 years of experience in radiology. According to perioperative CT or MRI, the radiologists focused on detecting tiny HCC. Follow-up CT and/or MRI scans, AFP test, and PET-CT were used to differentiate lesions with lipiodol deposition or high CT value if necessary. Any disagreement in the diagnosis was settled by consulting with other experienced radiologists in our department.

#### Percutaneous thermal ablation

CT-guided percutaneous thermal ablation included RFA and microwave ablation in our study. The anesthetic techniques consisted of a combination of local anesthesia (1 % lidocaine), intravenous benzodiazepine (1.0–2.5 mg of midazolam), and propofol (2–2.5 mg/kg). The dose of anesthetic was adjusted according to the intraoperative situation. Percutaneous ablation was performed according to our routine protocol. The parameters of ablation, including ablation power, ablation time, and number of electrodes, were adjusted according to the manufacturer’s instructions, operator’s experience, and intraoperative CT evaluation.

**Fig. 2:**
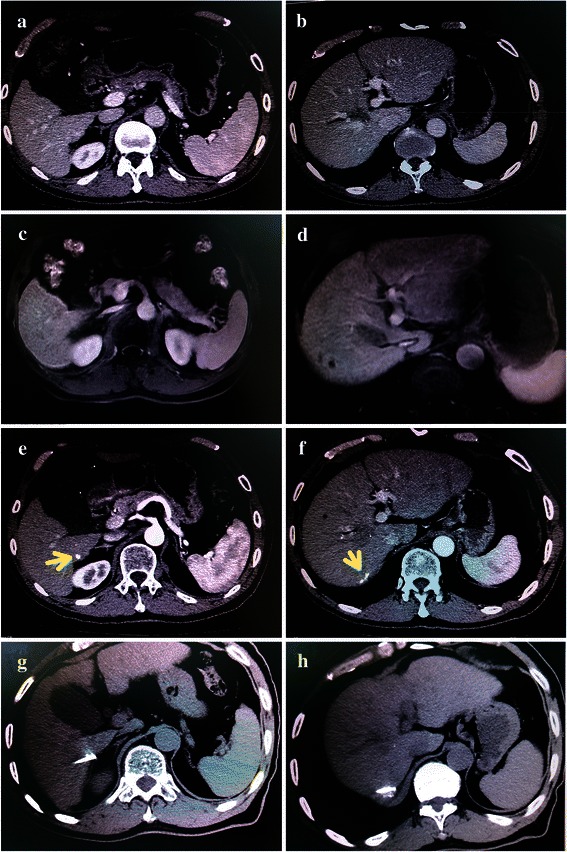
Perioperative imaging of patients with HCC. **a** and **b**, viable lesions are not shown in different segments of the liver via preoperative CT. **c** and **d**, it is very difficult to locate viable lesions in segments 6 and 7 of the liver via preoperative magnetic resonance imaging. **e** and **f**, two additional tiny lesions with lipiodol depositions were detected in segments 6 and 7 of the liver (*arrows*) by using lipiodol CT after TACE. **g** and **h**, two cancerous lesions were treated with radiofrequency ablation

For tumors more than 5 cm in diameter, we usually chose microwave ablation for HCC treatment; for tumors less than or equal to 5 cm, we used RFA. Most patients (78.9 %, 225 of 285) had cancerous lesions less than or equal to 5 cm in diameter. Complete ablation was assessed using intraoperative and postoperative contrast-enhanced CT (Fig. 2), and additional ablation was added if necessary. Five patients received palliative ablation in the combination group due to multiple lesions in the liver that were detected via subsequent lipiodol CT.

#### Hepatic resection

Hepatic resection was carried out under general anesthesia, and 4 experienced surgeons at Sun Yat-sen University Cancer Center performed these procedures. Intraoperative ultrasound was routinely used to evaluate other possible tumors, the remnant liver, and the possibility of a negative resection margin; the surgeon then searched for new lesions. Various methods of hepatic resection were adopted according to the scenario. After the operation, pathologic examination was performed for all excised specimens to confirm diagnosis and complete resection, which was considered the absence of microscopic tumor invasion and negative resection margins. Three patients were found to have incomplete resections due to new lesions detected during hepatectomy.

The treatment principal after recurrence was similar between the two groups. Hepatectomy, TACE, percutaneous thermal ablation, or conservative treatment was selectively performed in cases of recurrence depending on tumor location, tumor size, tumor number, liver function, and the general condition of the patient. For early recurrent HCC, either hepatectomy or RFA was performed according to the Barcelona Clinic Liver Cancer staging classification [12]. For unresectable recurrent HCC, palliative TACE, thermal ablation, or best supportive treatment was given. Recurrent patients who were not treated according to the above treatment principals were excluded from the surgery group.

Overall survival (OS) was measured from the date of diagnosis of HCC to the date of death or last follow-up. Recurrence-free survival (RFS) was defined as the interval between the time of diagnosis of HCC to the last follow-up or the time when a recurrent/residual tumor was diagnosed. Patients lost to follow-up were censored on the last follow-up day. The last follow-up date was March 1, 2014.

### Statistical analysis

The categorical characteristics were summarized and are presented as proportion and frequencies. The continuous variables are expressed as the mean values ± standard deviation, median values, or number of patients. Continuous data were compared between the two groups by using the independent *t* test, and categorical variables were compared by using Pearson’s chi-square test or Yates’s correction for continuity. Survival was calculated by using the Kaplan-Meier method and compared by using the log-rank test. A two-tailed *P* value of <0.05 was considered statistically significant. All statistical calculations were performed with SPSS software version 18.0 (SPSS, Chicago, IL, USA). The independent prognostic factors in predicting survival were assessed by using a multivariate Cox proportional hazards regression analysis.

## Results

### Survival analysis

**Fig. 3:**
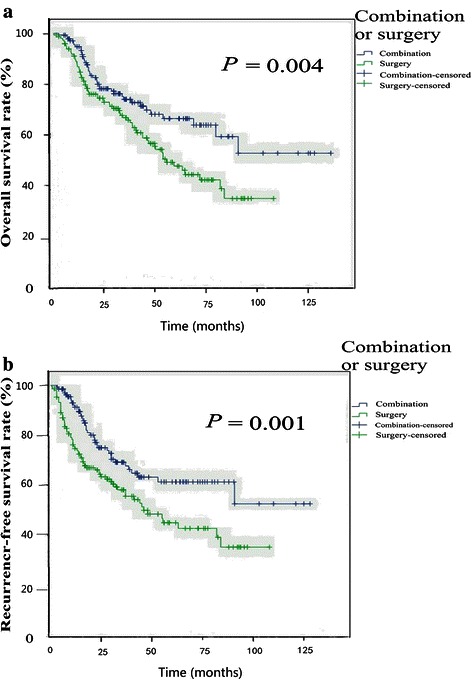
Comparison of survival between the combination group and surgery group of HCC patients. **a**, the combination of TACE and percutaneous thermal ablation improved the overall survival of patients with HCC compared with hepatectomy. **b**, the combination of TACE and percutaneous thermal ablation improved the recurrence-free survival of patients with HCC compared with hepatectomy

The mean follow-up duration was 43.4 ± 25.9 (median, 39.1) months, and 13 patients were lost to follow-up. The 1-, 3-, and 5-year OS rates were 95 %, 74 %, and 67 % for the combination group and 88 %, 66 %, and 47 % for the surgery group, respectively (*P* = 0.004). The 1-, 3-, and 5-year RFS rates were 92 %, 69 %, and 61 % for the combination group and 75 %, 58 %, and 44 % for the surgery group, respectively (*P* = 0.001) (Fig. 3).

In the multivariate analysis, treatment allocation (hazard ratio [HR] = 1.810, 95 % confidence interval [CI] = 1.215–2.697, *P* = 0.004), albumin level (HR = 1.819, 95 % CI = 1.233–2.685, *P* = 0.003), tumor size (HR = 1.732, 95 % CI = 1.160–2.587, *P* = 0.007), and tumor number (HR = 1.401, 95 % CI = 1.143–1.718, *P* = 0.001) were independent prognostic factors for OS (Table 2). Treatment allocation (HR = 1.999, 95 % CI = 1.341–2.980, *P* = 0.001), albumin level (HR = 1.723, 95 % CI = 1.167–2.543, *P* = 0.006), tumor size (HR = 1.877, 95 % CI = 1.255–2.806, *P* = 0.002), and tumor number (HR = 1.359, 95 % CI = 1.114–1.658, *P* = 0.002) were independent prognostic factors for RFS (Table 2).

**Table 2:** Univariate analysis of overall survival (OS) and recurrence-free survival (RFS) of the 285 HCC patients

Variable	Number of patients	OS (months)	*P*	RFS (months)	*P*
Age			0.879		0.921
<65 years	238	79.4 ± 4.6		72.4 ± 4.6	
≥65 years	47	77.9 ± 8.6		73.4 ± 9.4	
Sex			0.163		0.141
Male	262	83.0 ± 4.3		76.4 ± 4.2	
Female	23	54.0 ± 7.2		47.5 ± 8.1	
Cirrhosis			0.911		0.806
Yes	250	80.4 ± 4.4		73.7 ± 4.3	
No	35	67.6 ± 8.3		64.1 ± 8.8	
Treatment allocation			***0.012***		***0.004***
Combination^a^	137	91.2 ± 6.1		84.1 ± 6.0	
Surgery	148	61.7 ± 3.8		57.1 ± 4.3	
Tumor number			***<0.001***		***<0.001***
1	128	80.4 ± 5.0		66.7 ± 3.9	
2	93	73.3 ± 6.8		69.8 ± 7.2	
3	49	82.2 ± 9.5		75.3 ± 8.6	
4	15	31.8 ± 10.0		26.6 ± 7.7	
CA19-9			0.088		0.093
Positive	113	71.0 ± 5.8		68.0 ± 6.1	
Negative	172	84.1 ± 5.5		74.1 ± 4.9	
Alpha fetoprotein			***0.004***		***0.002***
≥400 ng/mL	79	60.6 ± 6.7		56.6 ± 7.1	
<400 ng/mL	206	87.2 ± 4.8		79.2 ± 4.5	
Albumin			***0.003***		***0.014***
≥40 g/L	174	87.4 ± 5.4		76.8 ± 4.9	
<40 g/L	111	65.3 ± 5.9		62.5 ± 6.2	
Prothrombin time			***0.018***		***0.011***
≤13.5 s	217	85.0 ± 4.9		78.2 ± 4.8	
>13.5 s	68	63.0 ± 6.8		58.2 ± 7.4	
Platelet count			0.417		0.604
≥100 × 10^9^/L	200	82.5 ± 4.9		74.0 ± 4.6	
<100 × 10^9^/L	85	72.1 ± 6.9		9.0 ± 7.5	
Hemoglobin			0.456		0.239
≥120 × 10^12^/L	247	81.6 ± 4.4		75.5 ± 4.2	
<120 × 10^12^/L	38	67.9 ± 9.3		51.6 ± 7.9	
Maximal tumor size			***0.009***		***0.004***
≤3 cm	130	95.0 ± 5.4		88.2 ± 5.3	
>3 cm	155	65.8 ± 4.8		56.1 ± 4.3	
Hypersplenism			0.941		0.668
Yes	144	79.4 ± 6.2		73.6 ± 5.9	
No	141	76.0 ± 5.0		71.8 ± 5.4	

All values are presented as mean ± standard deviation. ^a^Combination treatment includes ablation and hepatectomy.

### Complications

Complications are shown in Table 3. More blood loss and longer hospital stay were observed in the surgery group than in the combination group. Major complications did not significantly differ between the two groups. Hemoglobin level decreased more significantly in the surgery group than in the combination group (*P* < 0.001).

**Table 3:** Procedure-related events in the two groups of HCC patients

Variable	Combination group	Surgery group	*P*
Major complications (cases [%])	2 (1.5 %)	3 (2.0 %)	0.716
Bleeding volume (mL)	19.4 ± 42.0	327.0 ± 322.8	*<0.001*
Transfusion (mL)	5.1 ± 45.9	31.4 ± 114.8	*0.013*
HB decrease (mL)	8.9 ± 15.0	18.3 ± 15.6	*<0.001*
Hospital stay (days)	11.5 ± 6.9	18.7 ± 4.9	*<0.001*

The italic numbers reflected *P* value which was < 0.05

### Additional tumors detected

**Fig. 4:**
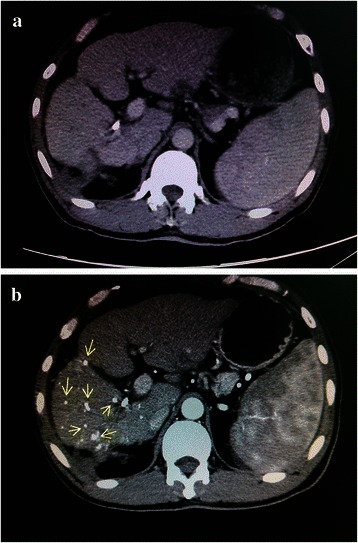
Multiple tiny lesions detected by lipiodol CT. **a**, no cancerous lesions were detected in contrast-enhanced CT prior to TACE. **b**, multiple tiny lesions with lipiodol depositions (as shown in *arrows*) were detected by using lipiodol CT after TACE in the same patient

Only 60 of the 137 patients in the combination group had sufficient imaging data to confirm tiny HCC in the liver. A total of 135 lesions with lipiodol deposition were diagnosed as new HCC lesions in 22 of 60 patients via lipiodol CT after TACE, as confirmed by radiologists in our team (Fig. 4). Twenty new lesions were found in 11 of 148 patients in the surgery group, as postoperatively confirmed via pathologic examination. Newly diagnosed tumors were ≤ 5 mm in diameter in the combination group whereas ≤ 10 mm in diameter in the surgery group.

## Discussion and conclusions

Surgery for resectable HCC has been widely used for many years. As new imaging techniques and technology advances, imaging-guided minimally invasive therapies for liver cancer have undergone rapid developments due to their efficacy and minimal invasiveness [13].

Our present study indicated that the combination of TACE and percutaneous thermal ablation improved the survival of patients with HCC compared with hepatectomy. The treatment modality was an independent prognostic factor for survival. TACE plus RFA can be used to achieve complete targeted HCC lesion necrosis; long-term survival has also been reported [14, 15]. TACE alone was reported to be comparable with RFA or hepatectomy for small single HCC [16]. Thus, TACE combined with RFA was possibly curative. Takuma *et al.* [9] found comparable OS rates between the two groups, and disease-free survival was superior in the surgery group than in the combination group. However, CT was performed 1 day after RFA, and there was no lipiodol CT imaging. Thus, additional tiny HCCs may not have been identified and treated in this study. Tashiro *et al.* [14] reported similar survival rates between the two therapies for patients with HCC ≤ 2 cm in diameter. However, for patients with a single Child-Pugh class A HCC larger than 2 cm in diameter, the disease-free survival time was significantly longer in the subgroup of hepatectomy than in the subgroup of RFA. Kim *et al.* [17] concluded that for a single HCC (2 to 5 cm in diameter), RFA combined with TACE resulted in a survival time similar to that of patients undergoing hepatectomy. For patients with early-stage HCC, Kagawa *et al.* [18] also stated that the OS rates of patients treated with RFA combined with TACE were similar to those of patients treated with hepatectomy.

We believe that combining TACE with percutaneous thermal ablation is beneficial. First, it is difficult to diagnose all of the tiny HCCs with routine contrast-enhanced CT or MRI. AFP level was significantly elevated in some patients; however, cancerous lesions cannot always be detected in imaging of these patients. We had to follow up until these lesions became apparent. When these tiny lesions coexist with benign lesions, it is more difficult to detect them using only AFP fluctuation. Second, during hepatectomy, it is difficult for the surgeon to detect very tiny lesions despite the routine use of intraoperative ultrasound. Some tiny HCCs were occasionally found in different segments, and these lesions were also difficult to take biopsy. Thus, they were less likely to be detected during hepatectomy. We only found 20 additional HCCs in the 148 patients in the surgery group. Third, subsequent lipiodol CT can be used to detect additional tiny HCCs with lipiodol deposition [6]. TACE combined with RFA was superior to RFA alone in prolonging patient survival, and the efficacy of TACE on HCC detection and treatment has been demonstrated [8]. Microvascular invasion with satellite lesions is an unfavorable prognostic factor for HCC [19]; however, tiny HCC and satellite lesions can be detected on lipiodol CT and then treated. TACE is useful for treating multiple lesions within different lobes, usually with incomplete necrosis. Complete necrosis can be achieved after thermal ablation, especially for local tumors. This explains why TACE combined with thermal ablation prolongs survival compared with hepatectomy. Fourth, HCC tends to relapse in a multi-centric manner. The vast majority of patients with HCC in China have hepatitis B virus infection and/or cirrhosis, which remain in the remnant liver after treatment. These factors limited the repeated use of hepatectomy for recurrent patients. Percutaneous thermal ablation, which is highly repeatable and less invasive to the liver parenchyma [20], more thoroughly preserves the liver parenchyma and is suitable for recurrent patients with poor performance status and cirrhosis.

New lesions were detected in only one-tenth of all patients and were found in 36.7 % of the 60 patients with complete data in our study. Additional cancerous lesions were not confirmed in the other 77 patients of the combination group; however, those lesions were ablated or closely followed up. Tiny lesions can be detected earlier and effectively treated with TACE and lipiodol CT in the combination group. Thus, this modality may provide a survival benefit. The surgery group had complete pathologic data; however, additional cancerous lesions were only identified in 11 of the 148 patients; any other non-detected lesions would lead to disease progression in the other 137 patients. Lipiodol CT was not performed in these patients, and many tiny lesions may not be identified. This may influence the efficacy of surgery because surgeons have difficulty in locating tiny HCCs in this circumstance. Thus, more tiny HCCs can be detected and treated by combining TACE with thermal ablation. Prior to resection or local ablation, neoadjuvant therapies are not recommended for HCC by some European experts [21]. According to our study, after TACE, lipiodol CT assists in the detection of additional cancerous lesions that were missed on routine contrast-enhanced CT or MRI imaging.

Tumor number and size were important factors in TNM staging and were independent prognostic factors in our study as well as in other studies [22–24]. There was no difference in preoperative maximal tumor size between two groups. In the combination group, more lesions were detected after TACE, and the total tumor number increased more substantially; thus, the mean tumor size became smaller. In the combination group, only 60 patients with sufficient imaging data were available for the discovery and confirmation of new lesions with lipiodol deposition; new tiny lesions in the other 77 patients were not confirmed as malignancies. In the surgery group, the pathologic data were complete and cancerous lesions were confirmed for all patients. However, lipiodol CT was not performed for these patients prior to surgery, thereby potentially leaving tiny HCCs undetected. Thus, the real mean size in the combination group was not definitively smaller than that in the surgery group.

Additionally, maximal tumor size affects TNM staging After TACE, the maximal tumor size in the combination group changed and the total tumor number increased. However, the maximal tumor size in the hepatectomy group was stable. Thus, we only provide a preoperative maximal tumor size for the survival analysis. Moreover, some cancerous lesions were undetected and unconfirmed in both groups. These lesions were later identified with differing methods, and only some additional lesions were confirmed. The median size of all of the tumors is thus not given.

After TACE, newly diagnosed lesions with lipiodol deposition on CT imaging cannot be verified via pathologic examination. However, these lesions may be cancerous and would thus require additional treatment or follow-up. Using MRI [25], contrast-enhanced CT, ultrasound, AFP test, and biopsy, accurate differential diagnoses were made for these additional lesions to exclude benign disease. However, a small subset of tiny lesions may be misdiagnosed as cancerous lesions and be over-treated. With TACE and lipiodol CT, most malignant lesions can be detected and treated. Cancerous lesions diagnosed preoperatively in the same patient were confirmed and effectively treated.

More precise techniques should be developed to further differentiate tiny HCC with higher diagnostic confidence. However, this is a retrospective study. A prospective multi-center randomized study is needed.

In conclusion, we indicated that the combination of TACE and percutaneous thermal ablation improved the survival of patients with HCC compared with hepatectomy. Lipiodol CT may assist in the detection of additional cancerous lesions in the liver.
